# Report of Diseases in the Universal Dispensary for Children St. Andrew's Hill, Doctors' Commons, from December 1, 1818, to June 1, 1819, Including a List of the Cases

**Published:** 1819-07-01

**Authors:** 


### VIII.

### *Report of Diseases in the Universal Dispensary for Children St. Andrew's Hill, Doctors' Commons, from December* 1, 1818, *to June* 1, 1819, *including a List of the Cases.*

| *Diseases.* | *No. of Cases.* | *Diseases.* | *No of Cases.* | *Diseases.* | *No. of Cases.* |
|---|---|---|---|---|---|
| Synochus . . . . | 64 | Chorea Sancti Viti . | 18 | Phthisis . . . . . | 2 |
| Typhus . . . . . | 4 | Hepatitis . . . . . | 2 | Icterus . . . . . . | 14 |
| Febris Intermittens | 8 | Enteritis . . . . . | 19 | Ascites . . . . . | 2 |
| —— Catarrhalis . . | 20 | Gastritis . . . . . | 12 | Pemphigus . . . . | 2 |
| Erysipelas . . . . . | 34 | Carditis . . . . . | 11 | Icthyosis . . . . . | 2 |
| Scarlatina . . . . . | 36 | Phrenitis . . . . . | 19 | Dysenteria . . . . | 20 |
| Morbilli . . . . . | 29 | Pleuritis . . . . . | 6 | Diarrhœa . . . . . | 47 |
| Varicella . . . . . | 23 | Pneumonia . . . . | 96 | Palpitatio Cordis . . | 6 |
| Urticaria . . . . . | 21 | Tussis . . . . . . | 24 | Bullæ . . . . . . | 4 |
| Roseola . . . . . | 10 | Ophthalmia . . . . | 26 | Anasarca . . . . . | 3 |
| Erythema . . . . . | 20 | Cholera Morbus . . | 26 | Vermes { Tænia | 2 |
| Strophulus . . . . | 20 | Rheumatismus . . . | 16 | Vermes { Ascarides | 12 |
| Cynanche Tonsillaris | 19 | Coryza Maligna . . . | 5 | Vermes { Lumbrici | 9 |
| ———— Maligna | 13 | Hydrothorax . . . . | 1 | Tormina . . . . . | 16 |
| ———— Trachealis | 34 | Paial sis . . . . . | 8 | Pyrosis . . . . . | 2 |
| ———— Parotidœa | 16 | Aphthæ . . . . . | 4 | Rachitis . . . . . | 15 |
| Pertussis . . . . . | 55 | Asthma . . . . . | 5 | Surgical cases . . . | 75 |
| Hydrocephalus Acutus | 28 | Syncope . . . . . | 2 | | |
| —— Chronicus . . | 30 | Hæmatemesis . . . | 2 | Total | 1130 |
| Convulsio . . . . . | 43 | Dyspepsia . . . . | 30 | | |
| Epilepsia . . . . . | 5 | Tabes Mesenterica . | 33 | | |

### MEDICAL.

Remarkable as the winter has been for its mildness, children have suffered under every kind of acute disease, with unusual severity. In the month of January, erysipelas, scarlatina, hooping-cough, and measles, were the reigning diseases, particularly scarlatina, which was frequently attended with, or followed by inflammation of the lungs, intestines, and brain.

Synochus was the most frequent form of fever. It occasionally degenerated into typhus; and at other times, it produced considerable congestion in the cerebral system, which ended in hydrocephalus. When this was the case, the children became comatose in the early stage of the fever, delirious, manifested the presence of acute pain in the head; then they were attacked with convulsions, which, when the disease proved fatal, recurred once in five or six hours. Several children expired in this state after twelve or fourteen days' duration of the fever.

Cholera morbus, enteritis, gastritis, pneumonia, and acute rheumatism, were also among the diseases of this month. Urticaria and varicella were likewise frequent and severe. The latter was accompanied with very high pyrexia, vomiting, acute pain in the head and back; the pustules were large, their bases widely inflamed, uniting with each other, and becoming confluent. These symptoms sometimes continued after the appearance of the eruption; and prevailed, for three or four days, with considerable severity, prior to its appearance. At the latter end of January, cases of pneumonia, combined with cynanche trachealis, began to be common; also cynanche tonsillaris and cynanche maligna, sometimes with, at others without, the scarlet efflorescence. The latter was no evidence either of the severity or mildness of cynanche tonsillaris or cynanche maligna; nor was the efflorescence necessary to prove an identity of the disease. It was perfectly obvious, that efflorescence was merely one of the appearances connected with cynanche, and not an essential character.

Affections of the trachea appeared under every modification in the month of February; but, in general, were of a very severe description. This disease was frequently preceded by catarrhal affections, and generally ended in pneumonia. Croup has not, for many years in London, prevailed so much as during the last six weeks. Four or five children have been attacked at the same time, in one family. In many children, croup, catarrhal affection, pneumonia, and convulsions, were simultaneous. Several infants of six weeks or two months old, were brought into the Dispensary with coryza, answering to the description of coryza maligna of authors. The pituitary membrane, throughout its whole extent, was in a state of active inflammation. A copious discharge, of a purulent nature, issued from the nostrils, and even from the mouth. Pneumonia and convulsions often succeeded this disease. Hydrocephalus acutus was, as usual, one of the most frequent diseases among the children at this Institution. It was, however, rarely idiopathic; not a primary, but a secondary disease. Two girls, about ten years of age, were attacked with well marked phrenitis. In young children there were many instances of this disease; but it appeared to be preceded by fever, or local inflammation of the thoracic or abdominal viscera.

Carditis was of frequent occurrence. Some of the patients under the influence of this disease were previously attacked with acute rheumatism. A boy, about eight years of age, was first attacked with acute rheumatism, from which he recovered; but, from imprudent exposure on the outside of a coach, on a wet day, was subsequently seized with pyrexia, acute pains, apparently pleuritic, cough, and extreme difficulty of breathing. After remaining a week in this situation, he lost all power of motion of the right side, experienced numbness of the lower extremities, syncope, and partial cold sweats; and he could only breathe, even with tolerable ease, when in an upright posture; very little palpitation of the heart was observable. At the end of three weeks from this second attack, he died and was opened. The thorax contained half a pint of fluid in its cavity; the pleura adhered in two or three places to the lungs; the heart was larger and heavier than usual, and all the valves were covered with red specks. The pericardium contained more fluid than usual. The abdominal viscera and brain were healthy. Is not rheumatism an inflammation of the muscular coats of small arteries? Is not rheumatism often combined with carditis?

A boy of ten years of age is in the Dispensary with icthyosis.

His constitution is evidently scrophulous; but his health is tolerably good. Two cases of pemphigus have occurred, and two of erythema nodosum. Chorea Sancti Viti, in its chronic form, has been frequent. Only two cases of intermittent fever have been met with in February.

Cynanche tonsillaris and maligna, cynanche trachealis, cholera morbus, enteritis, erysipelas, cynanche parotidœa, scarlatina, pneumonia, measles, and hooping-cough, all prevailed with severity in February: synochus was the reigning fever. Chronic diseases, as usual, were frequent; jaundice, dropsy, dyspepsy, tabes mesenterica, rickets, hydrocephalus chronicus, and cutaneous diseases.—Nor did the month of March produce much change in the general features of disease in children. Synochus, sometimes in conjunction with phrenitis, at others with gastritis and enteritis, and now and then modified with diarrhœa, cholera morbus, and icterus, was the common form of fever, both in March and in April; pure idiopathic synochus does not, in the Reporter's opinion, ever exist as a primary disease. Considerable pain and tension at the epigastric region, for instance, especially if attended with vomiting and constipation, during the existence of synochus, leave but little doubt of some disease having primarily affected the stomach; and if the pain and tension extend to the umbilicus and the hypochondria, or to either of these regions, the organs beneath them, it is probable, are in a state of disease previous to the developement of fever. The same remark holds good with respect to the head. Pain, giddiness, confusion of thought, dullness, are felt before pyrexia takes place. The more common form of fever in children is evidently synochus; and when it changes its character, it oftener verges to synocha than typhus. Unless the brain is primarily affected, in a very severe manner, the fever is rarely of a typhoid nature, and not then, until the disease has been of eight or ten days' duration.

Some few cases of vernal intermittent have occurred; all of which, with the exception of one, were mild, and effectually removed by small doses of the submuriate of mercury, administered night and morning. Pneumonia has diminished since March, in the frequency of its appearance, but not in severity; it is one of the most common, as well as one of the most fatal diseases of infancy, and very insidious in its approach, often existing without much pyrexia, and when the cough is slight. Nor is it unusual for pneumonia to exist with carditis. If the respiration of a child be quick, but particularly if it be laborious, and apparently painful; if the child wheezes, coughs occasionally, and is sleepless, it is dangerous to delay topical bleeding and active purging, even though there be scarcely any pyrexia. There is no acute disease to which children are liable, that is not instantly relieved by topical or general bleeding. When this remedy fails of its usual efficacy, it is either because it is not resorted to early enough, and to a sufficient extent, or because it is pushed too far, at an advanced period of the disease, when the child is exhausted, or an extensive change of structure produced.

In every Report, hydrocephalus forms a prominent feature, both as an acute and as a chronic disease. A brief statement of an extraordinary case of hydrocephalus congenitus will be interesting.—

From the period of this child's* birth to the present day, the head, which was much larger at the birth than natural, has rapidly increased, and has acquired such an extent as to now measure twenty-four inches round the chin and vertex, and the same number of inches round the forehead and occiput. Mr. Cox, the apothecary of the Institution has handed this measurement to the Reporter. All the sutures of the head are widely separated. The sagittal suture is at least an inch in width, and may be traced to the ossa nasi; the fontanel is nearly as broad as the palm of the hand. The coronal suture is half an inch wide, and the lambdoidal suture unusually open. Such is the weight of this enormous head, that the child is incapable of holding it upright; it falls back, and inclines to the right side. The shoulders are thrust up, and the dorsal vertebræ incurvated. The eyes are perpetually in motion; the pupils slightly dilated: vision apparently good. The child is perfectly sensible of its parents' frowns and caresses, and possessing the same degree of intellectual capacity as is common to children of that age; the parents think more. The complexion is healthy; the face plump, the body and limbs the same, all partaking, however, less of obesity than bloatedness. With a disease of the magnitude described, it will excite surprise to hear that the child's health has been uniformly good from its birth to the present time. It was the intention of the Reporter to have had the dura mater punctured by Mr. Dendy, one of the surgeons of the Institution; but he regrets to add, that the child's parents have taken it into the country, from a fear probably of the operation being attempted.

In the course of the three last months, erysipelas has been frequently seen; and, in many instances, in a very aggravated form, not only attacking the face and the body, about the pubes, but the lower extremities, principally at the upper and inner parts of the thighs, producing a continuity of the eruption with that in the region of the pubis. The vesications were large on the face and confluent, those on the thighs and pubis small and distinct. The œdematous swelling, which was of a very deep red colour, and tense to the touch, extended over the scalp, and often down to the shoulders. In one very severe case, the abdomen also was tense, exceedingly tender, and covered with numerous vesications. The Reporter could not divest himself of the opinion of the existence of peritoneal inflammation, in this instance, in conjunction with erysipelas. The child, who was two years of age, was violently convulsed, vomited frequently, and was delirious. Its pulse was

* Harriet Caxton, residing at Hackney, aged eleven months.

rapid and strong, the respiration quick, and the bowels confined. The disease had been of three days' duration when the child was brought to the Dipensary. Four leeches were ordered to be applied, two to the abdomen, and one behind each ear; and four more to be applied to the same parts the following day. The bowels were opened with ol. ricini, and two grains of the submuriate of mercury administered once in six hours, during three days, and one grain and a half of the pulv. ipec. co. given every night at bed-time. This plan succeeded. The disease yielded from the commencement, and the child entirely recovered under the use of diaphoretic and slightly aperient medicines.

Another child was brought to the Institution in a very advanced stage of erysipelas, and died three days after its admission: it was one year old; its face was covered with one continued black crust, which extended from ear to ear, and from the chin to the posterior part of the scalp. No vestige of a feature could be traced. From the eye-lids, which were glued together, a purulent fluid occasionally issued. From the nostrils, which were thick and large, flowed a similar fluid, in one continued stream; and from the angles of the mouth issued a brown fœtid ichor. The face exhibited the appearance of the face of a person in the last stage of small-pox of the worst description, before dissolution. In different parts of the body and extremities, erysipelatous blotches broke out. Local bleedings, evaporating lotions to the face, and small doses of the submuriate of mercury, in conjunction with the pulv. ipec. comp. produced a temporary amendment; but the child expired in a convulsion, with which it was attacked on the third day.

Cholera morbus has, at this season, been more frequent than usual, among children. It has now and then preceded phrenitis, and has been always accompanied with considerable pyrexia, sometimes with gastritis and enteritis: very young children were most often the subjects of it. The Reporter is convinced, that many children who are suddenly carried off in a fit, die from inflammation of the stomach, or the intestines. Enteritis in particular, with intus-susception, exists often, when a child's illness is attributed to irritation of the primæ viæ, owing to a dyspeptic state of the stomach and bowels. From some accidental cause, the inflammation undergoes a sudden and considerable increase, and the patient unexpectedly dies in a fit.—Dissection has shown, that children who had been first attacked with pneumonia, and who had recovered of this disease, had died of inflammation of the ileum, when death had been attributed to convulsion, from irritation merely of some other part primarily affected. This was placed beyond all doubt in one particular instance, where the brain was entirely free from disease; the heart sound; the lungs free from every appearance of recent disease; the upper portion of the ileum for about three inches was inflamed, of which there was no suspicion, as the child's bowels were thoroughly open, and who, with the exception of having slight pyrexia and vomiting, occasionally, appeared in all other respects to be regaining its health very fast.

Scarlatina has been generally mild. Varicella so severe as to be preceded by very high pyrexia for three days; delirium; incessant vomiting; acute pains in the back and head; jactitation; a hard and rapid pulse. Slight laxatives and saline diaphoretics were all that were necessary in scarlatina; varicella required local bleedings from the head and epigastric region, and active purging.

In several cases of phrenitis, attended with convulsions, and other fore runners of effusion into the brain, the Reporter has had the opportunity of carrying a general bleeding into effect with the greatest benefit. A child of two years old had nearly three ounces of blood drawn from the arm, after having had two fits, which had left it in a comatose state, with immediate relief. The fits did not return. The child became a convalescent from the moment it was bled. Upon another occasion, the external jugular was opened, and about the same quantity of blood drawn off; this case also ended well. The Reporter has no doubt of such vigorous treatment often averting hydrocephalus, unless previous exhaustion, combined with previous and more extended disease, renders it hazardous to resort to this active practice.

Measles have been mild, and by no means general. Hooping-cough, on the contrary, general and severe. Most of the cases brought to the Dispensary have been combined with pneumonia. In a boy of six years old, the lungs were condensed in large portions, slightly inflamed in others, and from some parts of the lungs, drops of pus were squeezed out. The heart, in this subject, was also inflamed. Local bleedings proved more serviceable in the treatment of hooping-cough than any other remedy; even in the milder cases they were highly useful; and if the submuriate of mercury does not remove hooping-cough, it always renders it more mild, by diminishing both the severity and the duration of the fit. Urticaria and roseola were among the exanthemata of most frequent occurrence.

Several well marked cases of rheumatism have been seen among children under three years of age. They required local bleedings, in addition to diaphoretics and mercurial purges for their removal. Dysentery, it will be observed in the catalogue of diseases, has been rather frequent. It has appeared both as a primary disease, and as a disease in connection with mesenteric disease. Chronic dysentery is more commonly met with among children than dysentery in its acute form, and is then nearly always connected with other visceral disease, usually of the mesentery and liver. Icterus, with fever and without fever, singly and conjoined with ascites, or tabes mesenterica, has been met with very frequently: paralysis of the lower extremities in eight instances. Of all the chronic affections, however, to which infancy is liable, dyspepsy is the most general; tabes mesenterica, and rickets, contribute also, in no small degree, to fill the Institution.

J. B. DAVIS, M D. Physician.

June 1st. 1819.

### SURGICAL.

The Surgical Diseases of Children, which have fallen under the Reporter's notice during the three last months, have consisted principally of scrophulous enlargements of the glands, scrophulous affections of the skin, the spine, and joints; ophthalmia, distortions from rickets, abscess, and cutaneous eruptions of different kinds: besides which, many local affections appearing in the form of tumour, inflammation and ulcer have occurred, in connection with constitutional symptoms; affections which have established a secondary disease, as difficult of removal as the primary one. Many instances of extensive cuticular inflammation, and ulcer, after fever and rheumatism have presented themselves; which, after baffling medical and surgical treatment, have yielded only to a change of air, and a well arranged dietetic system.

There is, at this time, an interesting Case, in the Dispensary, of a child who, after having had considerable incurvation of both tibiæ, the result of an attack of typhus, is, under the use of a nutritious diet, and the artificial support given to the tibiæ by instruments, fast recovering its health, and the form and functions of its legs.

A girl attends, with a strumous enlargement of the cervical glands. On the skin of the face, the small follicles inflame, suppurate, and coalesce, producing an ulcer, and corrugation of the integuments on healing. The case is remarkable, both for its long standing, and for the peculiarity of the eruption itself. It has resisted numerous remedies; but, at length, appears to be yielding to the use of a caustic solution and tonic medicines.

A boy has recovered from scrophulous ulceration of the sebaceous glands of the eye-lids and ophthalmia, by the use of the ungt. hydrarg. nitrat. and antimonial medicines.

A girl attends with tumours in the neck, accompanied with erysipelatous inflammation of the face, and an abscess at the internal angle of the left eye. The inflammation is removed, and the abscess healed; but the glands still remain enlarged. To these she is using ungt. hydrarg. dilut.

A child was brought with acute inflammation of the periosteum of the tibia, from contusion; with intense pain on pressure, and inability to move the limb. A large blister was laid over the gastrocnemii, after the application of leeches, and the tibia constantly bathed with. liq. ammon. acet. The child can now walk without experiencing any pain, and is likely to recover perfectly, without the occurrence of any exfoliation.

A case was presented of ophthalmia, with ulcers of the cornea in both eyes, and loss of vision. The right eye is quite restored, and the left nearly so. The ulcers are filled up, a very slight opacity only remaining. The remedies employed were p. rhei or hyd. submur. &c. Lotions of zinc and arg. nitrat. and the ungt. hydr. nitr. mit.

An obstinate cutaneous affection of the face, resembling the

"Lepra alphos," as delineated by Willan, frequently disappearing and recurring, with great constitutional debility, has existed in a girl for several years, with little amendment. The remedies which appear the most useful, are lotio hydrarg. oxymur. and ungt. picis. with the internal exhibition of ferrum tartarizatum.

A case of double cataract occurs, with great dilatation of the pupil, but not from mechanical distension, indicating a paralytic state of nerve, and forbidding the operation.

A child was admitted with curvature of the spine, attended with considerable febrile symptoms, and difficulty of breathing, owing to the lateral contraction of the thorax. A seton has been introduced in the back, and rest enjoined; but no good effect has been yet produced in this case.

A scrophulous child was brought, with extensive abscess under the "fascia lata," originating in an affection of the lumbar region, with a tumour also on the left shoulder, and on each ancle, accompanied with great emaciation. The infant is troubled with cough, and frequent convulsions. The tumour on the thigh has been twice punctured. The first operation (May 25th) was followed by the evacuation of two ounces of pus, and about the same quantity oozed from the orifice on the first day. The child is as well as it was before the operation, sleeps naturally, and does not appear to be debilitated by the discharge. On the repetition of the puncture (May 29th), two ounces more of fluid escaped, the integuments became flaccid, and the thigh was strapped with adhesive plaster. The cough is much better, and the convulsions seldom occur.

W. C. DENDY,
Surgeon to the Universal Dispensary for Children.

